# Dosing of lumbar spinal manipulative therapy and its association with escalated spine care: A cohort study of insurance claims

**DOI:** 10.1371/journal.pone.0283252

**Published:** 2024-01-05

**Authors:** Brian R. Anderson, James M. Whedon, Patricia M. Herman

**Affiliations:** 1 Palmer Center for Chiropractic Research, Palmer College of Chiropractic, Davenport, Iowa, United States of America; 2 Health Services Research, Southern California University of Health Sciences, Whittier, California, United States of America; 3 RAND Center for Collaborative Research in Complementary and Integrative Health, RAND Corporation, Santa Monica, California, United States of America; Ospedale Pediatrico Bambino Gesu, ITALY

## Abstract

**Objective:**

The objective of this study was to evaluate the relationship between three distinct spinal manipulative therapy dose groups and escalated spine care by analyzing insurance claims from a cohort of patients with low back pain.

**Methods:**

We compared three distinct spinal manipulative therapy dose groups (low = 1 SMT visits, moderate = 2–12 SMT visits, high = 13+ SMT visits), to a control group (no spinal manipulative therapy) regarding the outcome of escalated spine care. Escalated spine care procedures include imaging studies, injection procedures, emergency department visits, surgery, and opioid medication use. Propensity score matching was performed to address treatment selection bias. Modified Poisson regression modeling was used to estimate the relative risk of spine care escalation among three spinal manipulative therapy doses, adjusting for age, sex, retrospective risk score and claim count.

**Results:**

83,025 claims were categorized into 11,114 unique low back pain episodes; 8,137 claims had 0 spinal manipulative therapy visits, with the remaining episodes classified as low dose (n = 404), moderate dose (n = 1,763) or high dose (n = 810). After propensity score matching, 5,348 episodes remained; 2,454 had 0 spinal manipulative therapy visits with the remaining episodes classified as low dose (n = 404), moderate dose (n = 1,761), or high dose (n = 729). The estimated relative risk (vs no spinal manipulative therapy) for any escalated spine care was 0.45 (95% confidence interval 0.38, 0.55, *p <0*.*001*), 0.58 (95% confidence interval 0.53, 0.63, *p <0*.*001*), and 1.03 (95% confidence interval 0.95, 1.13, *p = 0*.*461*) for low, moderate, and high dose spinal manipulative therapy groups, respectively.

**Conclusions:**

For claims associated with initial episodes of low back pain, low and moderate dose spinal manipulative therapy groups were associated with a 55% and 42% reduction, respectively, in the relative risk of any escalated spine care.

## Introduction

Low back pain (LBP) has been among the leading causes of global disability for more than two decades [1], with a one-year prevalence of 38% [2]. There is data suggesting this prevalence has more than doubled over the past two decades [3]. A meta-analysis including twenty studies found that approximately 67% of Americans with LBP utilize healthcare services for management of their pain [4], resulting in treatment costs of approximately $87 billion per year [5]. The impact on both quality of life and direct and indirect costs ranks LBP in the “very critical” category (along with coronary heart disease and diabetes) when prioritizing research funding support [6].

Treatment guidelines for the initial management of LBP advocate for non-pharmacological and conservative care including education (reassurance of good prognosis), self-management (remaining active), exercise, and spinal manipulation [7–9]. Contrary to these guidelines, many patients are exposed to low-value, non-guideline-based healthcare with corresponding substantial increases in cost [10–13]. This escalation of unnecessary LBP-related interventions through routine early use of imaging studies, opioid medications, injection procedures, and surgery has dramatically increased in the past two decades [10–13].

While primary care physicians (PCPs) provide initial care for most LBP patients, chiropractic management is also commonly utilized. Spinal manipulation therapy (SMT) is the most common treatment offered by chiropractors and is a recommended treatment for both acute and chronic LBP [14, 15]. Short term pain relief associated with SMT is similar to non-steroidal anti-inflammatory drugs used in the treatment of LBP [16]. Ninety-two percent of patients who chose chiropractic treatment for an initial episode of LBP will choose chiropractic again (vs 49% for PCPs) for subsequent episodes [17]. This return rate indicates a high degree of perceived effectiveness and patient satisfaction.

Several studies have investigated the dose-response relationship between SMT and clinical outcomes for cervicogenic headache [18–20], chronic LBP [21, 22] and lumbar stenosis [23]. In general, greater SMT frequency was associated with improved outcomes. Associations between SMT and healthcare utilization for back pain have also been studied, and generally find significant reductions in imaging, surgery, opioid medications, injections, specialist visits and emergency department (ED) visits when compared to other forms of care [24–28]. The gap in the literature that our study fills is understanding the relationship between SMT dose and exposure to escalated spine care. This relationship has important implications for providers deciding on best practices and for policymakers determining insurance coverage for SMT, which is often limited to a specific number of visits per year.

The primary aim of this project was to evaluate the relationship between SMT dose during an episode of back pain and exposure to escalated spine care in the form of imaging studies, injection procedures, ED visits, surgery, and/or opioid medications. We tested the hypothesis that SMT dose is a significant predictor of exposure to escalated spine care interventions; the directionality is unknown due to a lack of prior literature in this area.

## Methods

We conducted a retrospective cohort study of a deidentified health insurance claims dataset spanning the years 2012–2018 from a single, self-insured, fortune-500 company. Prior to receiving the dataset, third party de-identification removed all protected health information, therefore patient consent was not required. The Institutional Review Board of Northern Illinois University provided a written exemption from review according to federal guidelines.

Our cohort was restricted to patients aged ≥18 with a specific primary diagnosis code related to back pain (S1 Table) who were current employees or family members covered by the employee health plan. Our unit of analysis was an episode of care, defined as a consultation or a series of consultations for LBP, preceded and followed by a 90-day claim free window [29]. We restricted our analysis to the initial episode, so each episode is associated with an individual patient. Depending on duration, these episodes may include acute, subacute, or chronic classifications.

### Primary predictor

We identified recipients of SMT by the presence of current procedural terminology (CPT) codes 98940–98942, which are documented as “Chiropractic Services” by Medicare [30]. Three distinct SMT groups were developed based on the number of SMT procedures during the episode; low (1 SMT), moderate (2–12 SMT), and high (13+ SMT), which were compared to a control group (no SMT). Several studies assisted in the development of the SMT dose groups: 1- A clinical prediction rule for positive response to SMT found that LBP patients with 4/5 criteria were classified as responders after one SMT visit [31, 32]; 2- Weigel et al. [33] indicates a cutoff of more than 12 SMT visits per year as high volume users; 3- Haas et al. [22] found dose-response saturation specific to pain and functional status for LBP at 12 SMT visits; 4- Stevans et al. [34] documented an average of 6 SMT visits per episode of a musculoskeletal related pain diagnosis. We used these studies, along with clinical experience, to guide the development of our dosing groups. All SMT visits were provided in outpatient, community-based clinics.

### Primary outcome

For the purposes of this study, any escalated spine care was defined as the use of one or more of the following LBP-related interventions (S2 Table) provides specific procedure codes and generic opioid names): imaging studies (Xray, MRI, CT scan), opioid medications, injection procedures (epidural, trigger point, nerve block), ED visits (Evaluation & Management codes 99281–99285), and back surgery (fusion, laminectomy, decompression, neurostimulator implantation). Opioid medication fills were imported from a separate pharmaceutical database and case-matched by unique member ID; they must have been filled within a 30-day window from the index visit to link use with the LBP episode under investigation.

### Additional variables

Age, sex, claim count, allowed reimbursement, and retrospective risk (R.Risk) score were included as covariates. According to Optum Inc, “Retrospective models use risk markers for an individual for a base year to measure cost risk for that same year, and can be used for risk adjustment of the conditions a member was actually treated for” [35]. We used age at first claim and mean R.Risk score, as this variable can change during the episode with additional claims (possible range 0–100). Claim count included the number of dates with an LBP-related claim during episode one. Allowed reimbursement included the amount reimbursed by the insurer during episode one. Mean episode duration (in months) was calculated and reported. This variable was not included as a covariate, as the reported values are estimates based on month and year (not day) provided in our dataset.

Additional detail on our methodology following the Strengthening the Reporting of Observational studies in Epidemiology and Reporting of Studies Conducted using Observational Routinely-collected Data (STROBE/RECORD) guidelines can be found in S3 Table [36, 37].

### Data analysis

Data were cleaned and organized via R [38], Microsoft SQL server and Microsoft Excel. We used SPSS [39] to conduct data analysis. Descriptive statistics were calculated for each SMT dose group by sex, age, R.Risk score, claim count, and escalated spine care procedure counts. We estimated the relative risk of any escalated spine care with a modified Poisson regression model [40] fit through generalized estimating equations using an unstructured covariance matrix. This model is adjusted for sex, age, claim count, and R.Risk score. The same model was utilized to estimate relative risk of individual spine care procedures (see primary outcome section). Propensity score matching (PSM) was implemented (greedy 1:1 nearest neighbor) to control for selection bias among treatment groups. The predictor variables age, gender, claim count, and retrospective risk score were used to match each subject in the low, moderate, and high SMT groups with a subject in the no SMT group, thereby creating a new dataset. Allowed cost was not included in any models, as cost and care escalation are strongly correlated.

## Results

**Fig 1:**
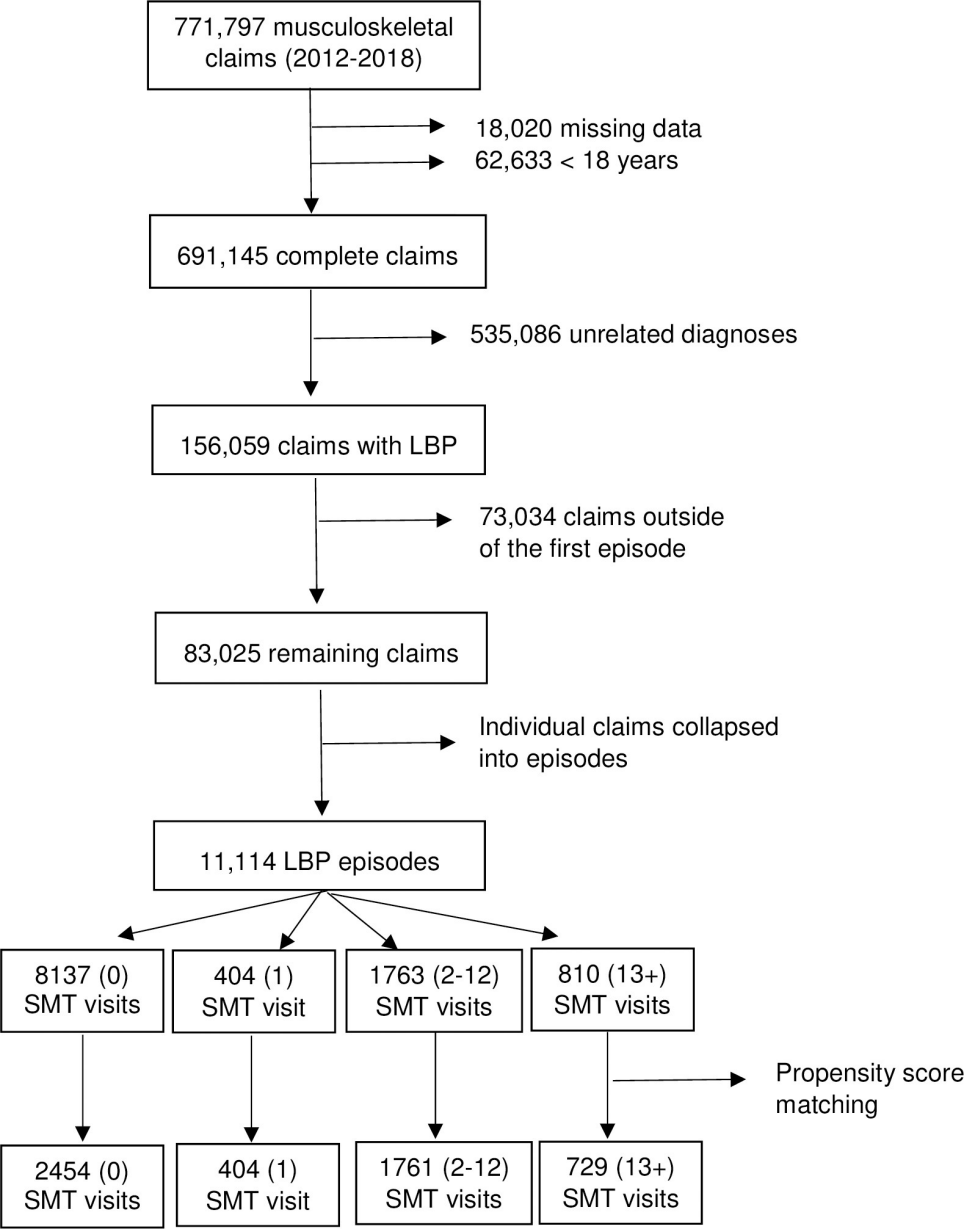
Cohort assembly.

The initial dataset consisted of 771,797 claims, spanning the years 2012 to 2018. After eliminating claims with missing data, those associated with individuals <18 years of age, those with anatomically unrelated diagnoses, and those outside of the first episode, 83,025 claims were categorized into 11,114 unique back pain initial episodes (Fig 1). PSM resulted in a 97% match rate and improved matching between the SMT and no SMT cohorts; descriptive characteristics of this matched cohort (n = 5,348) are presented in Table 1. The PSM process led to a decrease in the mean R.Risk score from 2.52 (before PSM- not shown) to 1.77 (after PSM) in the control group. The moderate dose cohort represented 61% of all SMT episodes, while the high dose cohort was associated with the highest mean allowed reimbursement ($1693), claim count (33), R.Risk score (2.02) and age (45). Mean episode duration ranged from 1.25 months in the low dose cohort (SD 1.33) to 3.65 months in the high dose cohort (SD 4.18). The overall rate of escalated spine care in our cohort was 39% (n = 2,099 episodes), with imaging being the most common procedure (30%) and surgery and ED visits being the least common (4%).

**Table 1:** Descriptive characteristics among SMT dose groups after propensity score matching.

	**No SMT**	**SMT (1)**	**SMT (2–12)**	**SMT (13+)**	**Overall**
**Overall n (%)**	2454 (46)	404 (8)	1761 (33)	729 (14)	5348 (100)
**Male n (%)**	1275 (52)	218 (54)	936 (53)	346 (48)	2775 (52)
**Mean age (SD)**	40 (13.5)	44 (13.5)	42 (13.5)	45 (13.5)	42 (13.5)
**Mean R.Risk (SD)**	1.77 (2.33)	1.84 (2.27)	1.87 (2.27)	2.02 (2.28)	1.84 (2.27)
**Mean claim count (SD)**	9 (32)	3(32)	7 (32)	33 (32)	11 (32)
**Mean Allowed (SD)**	953 (4557)	988 (4556)	589 (4556)	1693 (4562)	936 (4555)
**Mean episode duration in months (SD)**	1.93 (3.35)	1.25 (1.33)	1.60 (1.86)	3.65 (4.18)	2.01 (3.04)
**Escalated spine care (Episode count, % within cohort)**	**No SMT**	**SMT (1)**	**SMT (2–12)**	**SMT (13+)**	**Overall**
**Imaging**	789 (32)	69 (17)	416 (24)	326 (45)	1600 (30)
**Injection**	421 (17)	21 (5)	81 (5)	74 (10)	597 (11)
**Emergency Dept.**	168 (7)	2 (0.5)	24 (1)	12 (2)	206 (4)
**Surgery**	135 (6)	11 (3)	39 (2)	43 (6)	228 (4)
**Opioids**	260 (12)	14 (3)	69 (4)	67 (9)	410 (8)
**Any escalated care**	1163 (47)	86 (21)	482 (27)	368 (50)	2099 (39)

SMT = spinal manipulative therapy; R.Risk = retrospective risk score; IQR = interquartile range; Any escalated care = presence of 1 or more spine care procedure

The adjusted relative risk (RR) of any escalated spine care, along with individual spine care procedures was significantly decreased in the low and moderate SMT cohorts when compared to no SMT. This RR ranged from 0.08 [95% Confidence Interval (CI) 0.03, 0.33] in the low dose group for ED visits to 0.73 in the moderate dose group (CI 0.66, 0.81) for imaging studies. The high dose cohort was associated with an increased RR (1.33, CI 1.20, 1.48) for imaging studies, and a decreased RR for injection (0.54, CI 0.43, 0.68) and ED visits (0.27, RR 0.15, 0.48). There was a significant interaction between male sex and RR of surgery (+30%) and imaging (+11%) vs females. Finally, the R.Risk variable was directly associated with an increased risk of escalated spine care. All estimates were significant at p<0.001 (Table 2).

**Table 2:** Relative risk of escalated spine care among SMT dose groups.

	Imaging RR (95% CI)	Injection RR (95% CI)	ED RR (95% CI)	Surgery RR (95% CI)	Opioids RR (95% CI)	Any escalated care RR (95% CI)
**1 SMT**	**0.53 (0.43–0.67)**	**0.30 (0.19–0.45)**	**0.08 (0.02–0.33)**	**0.49 (0.27–0.90)**	**0.33 (0.20–0.56)**	**0.45 (0.38–0.55)**
**2–12 SMT**	**0.73 (0.66–0.81)**	**0.26 (0.21–0.33)**	**0.21 (0.14–0.32)**	**0.40 (0.28–0.57)**	**0.37 (0.29–0.48)**	**0.58 (0.53–0.63)**
**13+ SMT**	**1.33 (1.20–1.48)**	**0.54 (0.43–0.68)**	**0.27 (0.15–0.48)**	0.97 (0.70–1.36)	0.82 (0.63–1.06)	1.03 (0.95–1.13)
**Male**	**1.11 (1.02–1.20)**	1.12 (0.97–1.31)	0.85 (0.65–1.11)	**1.30 (1.001–1.68)**	1.01 (0.83–1.22)	1.06 (0.99–1.12)
**Age (years)**	1.002 (0.99–1.01)	**1.01 (1.008–1.02)**	**0.97 (0.96–0.98)**	**1.02 (1.01–1.03)**	1.004 (0.997–1.011)	0.999 (0.997–1.002)
**R.Risk**	**1.05 (1.04–1.06)**	**1.07 (1.04–1.09)**	**1.09 (1.05–1.13)**	**1.11 (1.09–1.14)**	**1.09 (1.06–1.12)**	**1.05 (1.04–1.06)**
**Claim count**	**1.001 (1.001–1.002)**	**1.002 (1.001–1.002)**	1.000 (0.999–1.002)	**1.002 (1.001–1.003)**	**1.002 (1.001–1.003)**	**1.001 (1.000–1.002)**

Reference group = No SMT; SMT = spinal manipulative therapy; ER = Emergency Department; R.Risk = retrospective risk score; IQR = interquartile range; Any escalated care = presence of 1 or more spine care procedures

## Discussion

Our hypothesis that SMT dose would be a significant predictor of escalated spine care exposure was confirmed. The low and moderate dose SMT groups were associated with a significant decreased risk of any escalated spine care, as well as each intervention studied. These associations in the high dose group were mixed: Increased risk of imaging; decreased risk of injection and ED visits; and no significant association with surgery, opioids, and any escalated spine care. Males were at significantly higher risk of surgery and imaging exposure, while increased R.Risk score was associated with all escalated spine care interventions.

Haas [22] evaluated four different doses (0, 6, 12, 18) of SMT over a six-week period on a variety of clinical outcomes in 400 LBP patients. A saturation of the dose-response relationship was found at 12 SMT sessions, where no additional benefit with more visits was observed. These results may have relevance to our study, as our high dose SMT group (13+) was associated with higher risk of imaging studies (vs no SMT and low and moderate dose SMT), and higher risk of injection and ED visits (vs low and moderate dose SMT). A number of studies investigating the role of chiropractic care on LBP related healthcare utilization confirm our findings. Fritz et al. [25] found that initiating LBP care with a chiropractor (vs primary care) decreased the odds for future advanced imaging (OR = 0.21, p-value = 0.001) and surgeon visits (OR = 0.13, p-value = 0.005), but increased care duration. Nelson et al. [26] measured the effects of insurance plans including vs excluding chiropractic coverage on utilization of advanced imaging, surgery, and plain-film radiographs in the management of neck and LBP. The group including (vs excluding) chiropractic benefits had a statistically significant decrease in utilization of surgery (-32%), advanced imaging (-23%) and plain film radiography (-8%). In a study evaluating nearly 15,000 episodes of LBP, individuals accessing chiropractic care were significantly less likely to record guideline-incongruent use of imaging, medications, and surgery [24]. A recent systematic review/meta-analysis found that spinal pain patients had a 64% reduction in odds of receiving opioid medications when accessing chiropractic care vs other types of care [27]. More recently, Whedon et al. [28] evaluated older patients with chronic LBP and found that those initiating care with SMT had significantly lower prevalence of spine care utilization vs those initiating care with opioid analgesic therapy.

Supporting our finding that males are at increased risk of spine surgery, a large multicenter database of spine surgery [41] documented a 60:40 Male:Female ratio. Other studies have confirmed that higher comorbidity scores (such as R.Risk) are associated with worse long term prognosis and increased healthcare utilization in patients with LBP [42].

Two studies evaluating the number of SMT-related visits for an episode of LBP over a 12 month period found a mean of 6.70 and 15.7, respectively [43, 44]. Davis et al. documented a mean of 8.5 all cause chiropractic visits per year utilizing data from the Medical Expenditure Panel Survey. The results of our study in combination with those of Haas et al., suggest 1–12 SMT-related visits for the management of LBP episodes.

## Limitations

Several limitations of our study should be discussed. First, our study did not distinguish between appropriate and inappropriate use of escalated spine care interventions; additional clinical information (such as severity and complexity of LBP) would help with this distinction. There are currently no adequate recommendations on how to subclassify SMT dosing. The authors used a combination of expert opinion (e.g., clinical experience) and indirect recommendations from the literature, which provides some guidelines on determining an “average” number of SMT visits. The study by Weigel et al [33] evaluated older adults while the study by Stevans et al. [34] was not specific to LBP, which may limit their applicability to our study methods. Alternative grouping methods of SMT dose, or the utilization of SMT as a continuous variable could result in different outcomes and should be further investigated. It should also be pointed out that while the provider of SMT cannot be determined in our database, several studies have identified that chiropractors provide the majority of SMT in the US [45, 46]. Selection bias is a factor when patients choose between different treatment options; we implemented propensity score matching methods to minimize this source of bias, but variables not included in the propensity match process may have impacted treatment selection. Finally, whether SMT is causally linked with spine care utilization, or if higher dose SMT is itself a component of escalated care that occurs with more complex back pain cases is unknown. There are also inherent limitations to analysis of administrative data, including unknown accuracy of billing codes, risk of unmeasured confounding variables, inclusion/exclusion errors, and the influence of health insurance coverage on the utilization of certain services [47].

## Conclusion

Our hypothesis that escalated spine care would vary depending on SMT dose was confirmed, and remained after adjusting for age, sex, retrospective risk score and claim count. Low and moderate dose SMT were consistently associated with lower risk of escalated spine care, while high dose SMT had mixed associations. The association between use of SMT and reduction in spine related healthcare utilization appears to be dose dependent. Our results indicate a need for further work in this area.

## Supporting information

S1 Table Included diagnosis codes (ICD-10). (DOCX)

S2 Table Included procedure codes (CPT), and opioid medications (generic names). (DOCX)

S3 Table STROBE/RECORD document. (DOCX)
