# Supplementary material for: Dosing of lumbar spinal manipulative therapy and its association with escalated spine care: A cohort study of insurance claims
Source: PLoS One. 2024 Jan 5;19(1):e0283252. doi: 10.1371/journal.pone.0283252 (PMC10769084; doi:10.1371/journal.pone.0283252)
Supplement: S1 Table — (DOCX) [file pone.0283252.s001.docx]

| lumbar ICD-9 (10) |  |
| --- | --- |
| 721.2 (M478.14) | Thoracic spondylosis without myelopathy |
| 721.3 (M478.14) | Lumbosacral spondylosis without myelopathy |
| 721.4 (M471.4, M471.6) | Thoracic or lumbar spondylosis with myelopathy |
| 721.5 (M482.0) | Kissing spine; Baastrup's syndrome |
| 721.6 (M481.0) | Ankylosing vertebral hyperostosis |
| 721.7 (M483.0) | Traumatic spondylopathy |
| 721.8 (M489) | Other allied disorders of spine |
| 721.9 (M489.19; M471.0) | Spondylosis of unspecified site |
| 722.1 (M512.6,-.7; M512.4-.5) | Displacement of thoracic or lumbar disk w/o myelopathy |
| 722.2 (M519) | Displacement of disk, site unspecified, w/o myelopathy |
| 722.5 (M513.4-.7) | Degeneration of thoracic or lumbar intervertebral disk |
| 722.6 (M513.4-.7) | Degeneration of intervertebral disk, site unspecified |
| 722.7 (M519, M500.0, M510.4-.7) | Intervertebral disk disorder with myelopathy |
| 722.8 (M961) | Postlaminectomy syndrome |
| 722.9 (*) | Other and unspecified disk disorder |
| 724.2-.9 (**) | Other and unspecified disorders of back |
| 738.4 (M4300, M4310) | Acquired spondylolisthesis |
| 738.5 (M9983, M9984) | Other acquired deformity of back or spine |
| 739.2 (M9902) | Non-allopathic lesion- Thoracic region |
| 739.3 (M9903) | Non-allopathic lesion- Lumbar region |
| 739.4 (M9904) | Non-allopathic lesion- Sacral region |
| 839.2 (S33101A, S23101A) | Dislocation- Thoracic and lumbar vertebra, closed |
| 839.3 (S31000A,S33101A,S21209A,S23101A) | Dislocation- Thoracic and lumbar vertebra, open |
| 839.4 (S2320XA, S332XXA, S3339XA) | Dislocation- Other vertebra, closed |
| 846.0-.3 (S338XXA, S336XXA, S339XXA) | Sprains and strains of sacroiliac region |
| 847.1 (S23XXA, S238XXA) | Sprains and strains of thoracic |
| 847.2 (S335XXA) | Sprains and strains of lumbar region |
| 847.3 (S338XXA) | Sprains and strains of sacrum region |
| 847.4 (S338XXA) | Sprains and strains of coccyx region |
| Diagnostic codes are provided in both ICD-9 & (ICD-10) since the claims database contained both | |
| * M464.0, M51.9, M508.0, M509.0, M464.5, M518.4, M518.5, M464.7, M518.6, M518.7 | |
| ** M480.0, M480.4, M480.6, M480.8, M546, M543.0, M541.4-.7, M548.9, M54.9, M432.7-.8, M532X7, M53.3, M532X8, M540.8, M438X9, M53.9 | |

**S1 Table- Included diagnosis codes**
