# Supplementary material for: Dosing of lumbar spinal manipulative therapy and its association with escalated spine care: A cohort study of insurance claims
Source: PLoS One. 2024 Jan 5;19(1):e0283252. doi: 10.1371/journal.pone.0283252 (PMC10769084; doi:10.1371/journal.pone.0283252)
Supplement: S2 Table — (DOCX) [file pone.0283252.s002.docx]

**S2 Table- Procedure codes and opioid medications included**

| \| **Imaging CPT codes** \| \| --- \| \| 72110 X-RAY EXAM OF LOWER SPINE \| \| 72100 X-RAY EXAM L-S SPINE 2/3 \| \| 73510 X-RAY EXAM OF HIP \| \| 73522 X-RAY EXAM HIPS BI 3-4 \| \| 72170 X-RAY EXAM OF PELVIS \| \| 72020 X-RAY EXAM OF SPINE \| \| 72114 X-RAY EXAM L-S SPINE BENDING \| \| 72010 X-RAY EXAM OF SPINE \| \| 72202 X-RAY EXAM SACROILIAC \| \| 76140 X-RAY CONSULTATION \| \| 72120 X-RAY EXAM OF LOWER SPINE \| \| 72265 CONTRAST X-RAY LOWER SPINE \| \| 72082 X-RAY EXAM ENTIRE SPI 2/3 VW \| \| 72220 X-RAY EXAM SACRUM \| \| 72080 X-RAY EXAM THORACOLMB 2/> VW \| \| 73523 X-RAY EXAM HIPS BI 5/> \| \| 72270 CONTRAST X-RAY SPINE \| \| 73501 X-RAY EXAM HIP UNI 1 VIEW \| \| 72090 X-RAY EXAM OF TRUNK SPINE \| \| 72148 MRI LUMBAR SPINE W/O DYE \| \| 72158 MRI LUMBAR SPINE W/O & W/DYE \| \| 76800 US EXAM SPINAL CANAL \| \| 72131 CT LUMBAR SPINE W/O DYE \| \| 76377 3D RENDERING W/POSTPROCESS \| \| 76942 ECHO GUIDE FOR BIOPSY \| \| 72132 CT LUMBAR SPINE W/DYE \| \| 76376 3D RENDER W/O POSTPROCESS \| \| 76000 FLUOROSCOPE EXAMINATION \| \| 72275 EPIDUROGRAPHY \| \| 72133 CT LUMBAR SPINE W/O & W/DYE \| \| 74176 CT ABD & PELVIS \| \| 72195 MRI PELVIS W/O DYE \| \| 72149 MRI LUMBAR SPINE W/DYE \| \| 74150 CT ABDOMEN W/O DYE \| \| 74176 CT ABD & PELVIS W/O CONTRAST \| \| 74170 CT ABDOMEN W/O & W/DYE \| \| 76937 US GUIDE VASCULAR \| \| 72197 MRI PELVIS W/O & W/DYE \| \| 72193 CT PELVIS W/DYE \| | \| **Injection CPT codes** \| \| --- \| \| 96372 THER/PROPH/DIAG INJ SC/IM \| \| 62311 INJECT SPINE L/S (CD) \| \| 64483 INJ FORAMEN EPIDURAL L/S \| \| 64484 INJ FORAMEN EPIDURAL ADD-ON \| \| 27096 INJECT SACROILIAC JOINT \| \| 64493 INJ PARAVERT F JNT L/S 1 \| \| 64494 INJ PARAVERT F JNT L/S 2 \| \| 20553 INJECT TRIGGER POINTS 3/> \| \| 64495 INJ PARAVERT F JNT L/S 3 \| \| 20552 INJ TRIGGER POINT 1/2 MUSCL \| \| 20553 INJECT TRIGGER POINTS =/> 3 \| \| 62290 NJX PX DISCOGRAPHY LUMBAR \| \| 62304 MYELOGRAPHY LUMBAR \| \| 62323 NJX INTERLAMINAR LMBR/SAC \| \| 62284 INJECTION FOR MYELOGRAM \| \| 64490 INJ PARAVERT F JNT C/T 1 \| \| 64491 INJ PARAVERT F JNT C/T 2 \| \| 62322 NJX INTERLAMINAR LMBR/SAC \| \| 64640 INJECTION TREATMENT OF NERVE \| \| 64445 N BLOCK INJ SCIATIC SNG \| \| 64450 N BLOCK OTHER PERIPHERAL \| \| 96374 THER/PROPH/DIAG INJ \| \| 62273 INJECT EPIDURAL PATCH \| \| 62327 NJX INTERLAMINAR LMBR/SAC \| \| 62319 INJECT SPINE W/CATH L/S \| \| 62305 MYELOGRAPHY LUMBAR \| \| 96365 THER/PROPH/DIAG IV INF INIT \| \| 96375 TX/PRO/DX INJ NEW DRUG ADDON \| \|  \| \|  \| \|  \| \|  \| \|  \| \|  \| \|  \| \|  \| \|  \| \|  \| \|  \| \|  \| \|  \| \|  \| \|  \| \|  \| \|  \| \|  \| \|  \| \|  \| \|  \| \|  \| \|  \| \|  \| \|  \| \|  \| \|  \| \|  \| \|  \| \|  \| \|  \| \|  \| \|  \| \|  \| \|  \| \|  \| \|  \| | \| **Surgical CPT codes** \| \| --- \| \| 63030 LOW BACK DISK SURGERY \| \| 22558 LUMBAR SPINE FUSION \| \| 22853 INSJ BIOMECHANICAL DEVICE \| \| 460 SPINAL FUSION EXCEPT CERVICAL \| \| 22612 LUMBAR SPINE FUSION \| \| 22840 INSERT SPINE FIXATION DEVICE \| \| 63042 LAMINOTOMY SINGLE LUMBAR \| \| 63035 SPINAL DISK SURGERY ADD-ON \| \| 64635 DESTROY LUMB/SAC FACET JNT \| \| 64636 DESTROY L/S FACET JNT ADDL \| \| 22633 LUMBAR SPINE FUSION COMBINED \| \| 22851 APPLY SPINE PROSTH DEVICE \| \| 22830 EXPLORATION OF SPINAL FUSION \| \| 63047 REMOVE SPINE LAMINA 1 LMBR \| \| 63048 REMOVE SPINAL LAMINA ADD-ON \| \| 520 BACK & NECK PROC EXC SPINAL FUSION \| \| 20930 SP BONE ALGRFT MORSEL ADD-ON \| \| 22842 INSERT SPINE FIXATION DEVICE \| \| 63056 DECOMPRESS SPINAL CORD \| \| 63057 DECOMPRESS SPINE CORD ADD-ON \| \| 518 BACK & NECK PROC EXC SPINE FUSION \| \| 22845 INSERT SPINE FIXATION DEVICE \| \| 519 BACK & NECK PROC EXC SPINAL FUSION \| \| 20937 SP BONE AGRFT MORSEL ADD-ON \| \| 22849 REINSERT SPINAL FIXATION \| \| 22852 REMOVE SPINE FIXATION DEVICE \| \| 22585 ADDITIONAL SPINAL FUSION \| \| 22614 SPINE FUSION EXTRA SEGMENT \| \| 455 COMBINED ANT/POST SPINAL FUSION \| \| 69990 MICROSURGERY ADD-ON \| \| 20931 SP BONE ALGRFT STRUCT ADD-ON \| \| 22533 LAT LUMBAR SPINE FUSION \| \| 22534 LAT THOR/LUMB ADDL SEG \| \| 64714 REVISE LOW BACK NERVE(S) \| \| 63044 LAMINOTOMY ADDL LUMBAR \| \| 22634 SPINE FUSION EXTRA SEGMENT \| \| 63655 IMPLANT NEUROELECTRODES \| \| 63685 INSRT/REDO SPINE N GENERATOR \| \| 63012 REMOVAL OF SPINAL LAMINA \| \| 22899 SPINE SURGERY PROCEDURE \| \| 63012 REMOVE LAMINA/FACETS LUMBAR \| \| 22850 REMOVE SPINE FIXATION DEVICE \| \| 459 SPINAL FUSION EXCEPT CERVICAL W MCC \| \| 22630 LUMBAR SPINE FUSION \| \| 63650 IMPLANT NEUROELECTRODES \| \| 20936 SP BONE AGRFT LOCAL ADD-ON \| \| 63267 EXCISE INTRASPINAL LESION \| \| 454 COMBINED ANT/POST SPINAL FUSION \| \| 27279 ARTHRODESIS SACROILIAC JOINT \| \| 63661 REMOVE SPINE ELTRD PERQ ARAY \| \| 63688 REVISE/REMOVE NEURORECEIVER \| \| 22102 REMOVE PART LUMBAR VERTEBRA \| \| 63045 REMOVAL OF SPINAL LAMINA \| \| 63017 REMOVAL OF SPINAL LAMINA \| \| 63664 REVISE SPINE ELTRD PLATE \| \| 22632 SPINE FUSION EXTRA SEGMENT \| \| 63090 REMOVAL OF VERTEBRAL BODY \| \| 22846 INSERT SPINE FIXATION DEVICE \| \| 64772 INCISION OF SPINAL NERVE \| \| 64999 NERVOUS SYSTEM SURGERY \| \| 22214 REVISION OF LUMBAR SPINE \| \| 22848 INSERT PELV FIXATION DEVICE \| \| 22843 INSERT SPINE FIXATION DEVICE \| \| 63709 REPAIR SPINAL FLUID LEAKAGE \| \| 22810 FUSION OF SPINE \| | \| **Emergency Department E&M codes** \| \| --- \| \| 99281 \| \| 99282 \| \| 99283 \| \| 99284 \| \| 99285   \| **Opioid medications** \| \| --- \| \| ACETAMINOPHEN WITH CODEINE \| \| HYDROCODONE BITARTRATE \| \| TRAMADOL HCL \| \| BUPRENORPHINE \| \| BUPRENORPHINE HCL \| \| BUTALBIT/ACETAMIN/CAFF/CODEINE \| \| BUTORPHANOL TARTRATE \| \| CODEINE/BUTALBITAL/ASA/CAFFEINE \| \| FENTANYL \| \| HYDROMORPHONE HCL \| \| MEPERIDINE HCL \| \| MEPERIDINE HCL/PF \| \| METHADONE HCL \| \| MORPHINE SULFATE \| \| OXYCODONE HCL \| \| OXYMORPHONE HCL \| \| PENTAZOCINE HCL/NALOXONE HCL \| \| TAPENTADOL HCL \| \| \|  \| \|  \| \|  \| \|  \| \|  \| \|  \| \|  \| \|  \| \|  \| \|  \| \|  \| \|  \| \|  \| \|  \| \|  \| \|  \| \|  \| \|  \| \|  \| \|  \| \|  \| \|  \| \|  \| \|  \| \|  \| \|  \| \|  \| \|  \| \|  \| \|  \| \|  \| \|  \| \|  \| \|  \| \|  \| \|  \| \|  \| \|  \| \|  \| \|  \| \|  \| \|  \| \|  \| \|  \| \|  \| \|  \| \|  \| \|  \| \|  \| \|  \| \|  \| \|  \| \|  \| \|  \| \|  \| \|  \| \|  \| \|  \| \|  \| \|  \| |
| --- | --- | --- | --- | --- | --- | --- | --- | --- | --- | --- | --- | --- | --- | --- | --- | --- | --- | --- | --- | --- | --- | --- | --- | --- | --- | --- | --- | --- | --- | --- | --- | --- | --- | --- | --- | --- | --- | --- | --- | --- | --- | --- | --- | --- | --- | --- | --- | --- | --- | --- | --- | --- | --- | --- | --- | --- | --- | --- | --- | --- | --- | --- | --- | --- | --- | --- | --- | --- | --- | --- | --- | --- | --- | --- | --- | --- | --- | --- | --- | --- | --- | --- | --- | --- | --- | --- | --- | --- | --- | --- | --- | --- | --- | --- | --- | --- | --- | --- | --- | --- | --- | --- | --- | --- | --- | --- | --- | --- | --- | --- | --- | --- | --- | --- | --- | --- | --- | --- | --- | --- | --- | --- | --- | --- | --- | --- | --- | --- | --- | --- | --- | --- | --- | --- | --- | --- | --- | --- | --- | --- | --- | --- | --- | --- | --- | --- | --- | --- | --- | --- | --- | --- | --- | --- | --- | --- | --- | --- | --- | --- | --- | --- | --- | --- | --- | --- | --- | --- | --- | --- | --- | --- | --- | --- | --- | --- | --- | --- | --- | --- | --- | --- | --- | --- | --- | --- | --- | --- | --- | --- | --- | --- | --- | --- | --- | --- | --- | --- | --- | --- | --- | --- | --- | --- | --- | --- | --- | --- | --- | --- | --- | --- | --- | --- | --- | --- | --- | --- | --- | --- | --- | --- | --- | --- | --- | --- | --- | --- | --- | --- | --- | --- | --- | --- | --- | --- | --- | --- | --- | --- | --- | --- | --- | --- | --- | --- | --- | --- | --- | --- | --- | --- | --- | --- | --- | --- | --- | --- | --- | --- |
