# Supplementary material for: Dosing of lumbar spinal manipulative therapy and its association with escalated spine care: A cohort study of insurance claims
Source: PLoS One. 2024 Jan 5;19(1):e0283252. doi: 10.1371/journal.pone.0283252 (PMC10769084; doi:10.1371/journal.pone.0283252)
Supplement: S3 Table — (DOCX) [file pone.0283252.s003.docx]

STROBE/RECORD methods checklist ^1^

| Methods |  |
| --- | --- |
| Study design | Retrospective cohort analysis of insurance claims |
| Setting | Claims generated by insured members of a single, self-insured fortune 500 company covering approximately 50,000 individuals with a geographic location including various states in the southern US |
| Participants | Covered individuals aged >18 with a primary back pain related diagnosis. Patients with diagnoses related to cancer, fracture, infection or inflammatory spondylopathy are not included in the dataset. Included ICD-9 and ICD-10 diagnoses are listed in appendix 1 |
| Variables | 1. Episode of care= 90 day claim free period before and after first and last claim, respectively 2. Age= age at first visit of episode 1 3. Gender= male/female 4. Spinal manipulative therapy (SMT) = presence of any CPT code in the range 98940-98942 5. SMT dose cohorts   1 SMT=one CPT code (98940-98942) during episode 1  2-12 SMT= 2-12 CPT codes (98940-98942) during episode 1  13+= 13 or more CPT codes (98940-98942) during episode 1  Control group= No SMT   1. Imaging = CPT codes provided in Appendix 1 2. Injection= CPT codes provided in Appendix 1 3. Surgery= CPT codes provided in Appendix 1 4. Emergency Department= CPT codes provided in Appendix 1 5. Opioids= see Appendix 1 6. Escalated spine care = presence of any procedure (6-10) above 7. Retrospective risk score (R.Risk)= proprietary score calculated based on demographics, medical/pharmacy claims for the year prior to initial visit for episode 1. Mean R.Risk score was used for analysis. 8. Claim count = number of claims present for episode 1 9. Episode duration= # of months during which at least 1 claim was generated |
| Data sources/management | The dataset was made available via a collaboration with a private healthcare company called Integrative Musculoskeletal Care (IMC) based in Tallahassee FL. |
| Bias | Misclassification of treatment group is the most likely source of bias in our analysis. Our method of assigning an episode to the spinal manipulation group when evidence of this service was present at any time during the episode could potentially bias our results against spinal manipulation; we believe this strengthens the conclusions of our models.  Propensity score matching was utilized as a method of managing treatment selection bias  Other sources of potential bias are discussed in limitations |
| Study size | Adequate detail provided in Methods section |
| Quantitative variables | Table 1 report counts, mean/median values and ranges for each predictor based SMT dose cohort. |
| Statistical methods | Table 2 reports results of the modified Poisson regression models fit through generalized estimating equations with unstructured covariance matrix was employed for categorical outcome variables as described by Zou ^2-3^ to calculate relative risk of escalated spine care in each SMT dose group (vs no SMT) |
| Data access and cleaning methods | The cleaned dataset population is the study sample. Diagnosis codes (ICD-9, ICD-10) and Procedure code lists reduce the associated claims to those that are in-scope for IMC services provided to their clients (Dislocations, fractures, infections and inflammatory arthropathies are excluded). SM had full access to the deidentified dataset population; BA had access to a slightly more deidentified version of this dataset; JW and PH had no access to the dataset |
| Linkage | The data is person level linked by an encrypted unique member identifier. The quality of the linkage is co-verified by independent parties at both IMC and the SIE. Social security number, date of birth and gender are used to generate the encrypted IDs. |

1. Benchimol El et al. The Reporting of studies Conducted using Observational Routinely-collected health Data (RECORD) Statement. PLoS Med 2015, 12(10)
2. Zou, G. A Modified Poisson Regression Approach to Prospective Studies with Binary Data. American Journal of Epidemiology 2004, 159:7
3. Zou, G & Donner, A. Extension of the modified Poisson regression model to prospective studies with correlated binary data. Stat Methods Med Res 2011, 22:661
